# Infant Mortality in Europe

**Published:** 1920-11-06

**Authors:** 


### Infant Mortality in Europe.

### THE CASE OF THE WAR BABY.

Some extremely interesting and suggestive statistics are given by a writer in the *International Journal of Public Health* as to infant mortality in Europe in the twentieth century, from 1909 to 1918 inclusive. Throughout the last century the character and intensity of infant mortality underwent little variation, apart from passing fluctuations, due to the changing prevalence of epidemic diseases.

The toll of death during the first year of life was by the middle of the century from 15 per cent. in the well-advanced countries to 25 per cent. in the

**THE PUBLIC WELL-BEING—*(continued)*.**

less healthy regions, and practically no change was noted up to about 1901. Ireland and the Scandinavian countries only showed somewhat less severe conditions for infant life.

From the beginning of the new century the infant mortality decreased rapidly, interrupted only by the epidemic of summer diarrhœa of 1911, up to the outbreak of the European War. The remarkable saving of infant life, hitherto unparalleled in history, cannot be traced, says the writer, to a single cause, but has its biologic explanation in the decreased fecundity, the more favourable conditions of life, improved housing, the higher standard of education, and, last but not least, in the awakening interest in public-health problems and preventive medicine. It is hardly possible to measure the relative importance of each of the many new influences at work during this period.

When the war broke out the movement for the preservation of infant life was progressing rapidly, and it is gratifying that, even though the progress has been somewhat checked, most of the attained results have been maintained.

An examination of the tables presented from 1909 to 1918 shows that Norway and Australia have the best records, and Bavaria, Prussia, and Spain the worst. The lowest infantile death-rate per 1,000 was achieved in 1917 by Australia with a rate of fifty-six, which rose in the following year to fifty-nine. The highest in the period under review was recorded in Bavaria with a rate of 223 in 1911, while it topped the 200 mark in 1909, 1910, and 1918. In England the highest death-rate was 130 in 1911, and the lowest 91 in 1916. It is generally believed that the tiny babies have suffered particularly from the war, but this is not the case Children from two to five years of age show an increased mortality, although less so than old people, but the breasted infants are the last to suffer from the misery of war. The tables disclose that the year 1915 was generally unfavourable, although less so than 1911, but that 1916 is among the best years on record. Great Britain, Holland, and the Scandinavian countries have maintained the low infant mortality rates of the pre-war years. The rates for France are somewhat higher, though not nearly so high as during the first decade of the century. Switzerland has succeeded in materially decreasing her infant mortality, while no impairment is remarked in Germany. No recent data are available for the countries in Eastern Europe.

Data are not available as yet for the causes of infantile mortality during the war period, except for Great Britain and Holland. In England very little change has occurred beyond the fact that the diarrhœa mortality was reduced from 14.8 in 1912-14 to 11.5 during the period 1915-18. Even allowing for different methods of classification, it is evident that a further great saving of infant life is possible. In Australia the infant mortality, as we have already indicated, was reduced to fifty-six per 1,000 living births in 1917, and of the 7,266 deaths under one year 3,965, or 54 per cent., were due to malformation, congenital debility, and similar causes for which no prevention is possible. In other countries a much higher proportion of the deaths are due to preventable causes, and there is thus hope, as the statistician suggests, that the reduction of the infant mortality, which the war has interrupted, may be continued to a considerable extent. It would be an advantage in the interests of public health if these very valuable figures could be given a much wider publicity and subjected to a careful analysis.

